# Supplementary material for: Innovative practice of sustainable digital signal processing education
Source: PLoS One. 2026 Jan 16;21(1):e0340806. doi: 10.1371/journal.pone.0340806 (PMC12810817; doi:10.1371/journal.pone.0340806)
Supplement: Attachment — Submitted filename: Response to Reviewers.docx [file pone.0340806.s005.docx]

**Response to Reviewers**

Journal Requirements:

When submitting your revision, we need you to address these additional requirements.

[1.Please](https://1.please/) ensure that your manuscript meets PLOS ONE's style requirements, including those for file naming. The PLOS ONE style templates can be found at

<https://journals.plos.org/plosone/s/file?id=wjVg/PLOSOne_formatting_sample_main_body.pdf> and

[https://journals.plos.org/plosone/s/file?id=ba62/PLOSOne_formatting_sample_title_authors_affiliations.pdf](https://track.editorialmanager.com/CL0/https:%2F%2Fjournals.plos.org%2Fplosone%2Fs%2Ffile%3Fid=ba62%2FPLOSOne_formatting_sample_title_authors_affiliations.pdf/1/010f019a913a1680-8cd1e0e0-e977-4aa1-bc15-eb624c9dccca-000000/TY_H4bbyihGjnV5uarad-cypdddPstglK44fneJL5GQ=237)

**We have chected all our manuscripts, they all meet PLOS ONE's style requirements.**

2. Please note that funding information should not appear in any section or other areas of your manuscript. We will only publish funding information present in the Funding Statement section of the online submission form. Please remove any funding-related text from the manuscript.

**we have remove the funding-related text from the manuscript.**

3. We note that the grant information you provided in the ‘Funding Information’ and ‘Financial Disclosure’ sections do not match.

When you resubmit, please ensure that you provide the correct grant numbers for the awards you received for your study in the ‘Funding Information’ section.

**We have revised the information.**

4. We note that Figure 3 in your submission contain copyrighted images. All PLOS content is published under the Creative Commons Attribution License (CC BY 4.0), which means that the manuscript, images, and Supporting Information files will be freely available online, and any third party is permitted to access, download, copy, distribute, and use these materials in any way, even commercially, with proper attribution. For more information, see our copyright guidelines: <http://journals.plos.org/plosone/s/licenses-and-copyright>.

**we have removed the Figure 3.**

5. We note that Figure 4 includes an image of a participant in the study.

As per the PLOS ONE policy (<http://journals.plos.org/plosone/s/submission-guidelines#loc-human-subjects-research>) on papers that include identifying, or potentially identifying, information, the individual(s) or parent(s)/guardian(s) must be informed of the terms of the PLOS open-access (CC-BY) license and provide specific permission for publication of these details under the terms of this license. Please download the Consent Form for Publication in a PLOS Journal (<http://journals.plos.org/plosone/s/file?id=8ce6/plos-consent-form-english.pdf>). The signed consent form should not be submitted with the manuscript, but should be securely filed in the individual's case notes. Please amend the methods section and ethics statement of the manuscript to explicitly state that the patient/participant has provided consent for publication: “The individual in this manuscript has given written informed consent (as outlined in PLOS consent form) to publish these case details”.

If you are unable to obtain consent from the subject of the photograph, you will need to remove the figure and any other textual identifying information or case descriptions for this individual."

**We have removed the figures and any other textual identifying information.**

6. Please include captions for your Supporting Information files at the end of your manuscript, and update any in-text citations to match accordingly. Please see our Supporting Information guidelines for more information: [http://journals.plos.org/plosone/s/supporting-information](http://track.editorialmanager.com/CL0/http:%2F%2Fjournals.plos.org%2Fplosone%2Fs%2Fsupporting-information/1/010f019a913a1680-8cd1e0e0-e977-4aa1-bc15-eb624c9dccca-000000/xTBfQPRikdurM1YSUWNhMTec-SbNBt7tbs7_JjuTr54=237).

**We have added a title to our supporting information.**

7. Thank you for providing your underlying data as Supporting Information.

We note that the data set contains text or data that is not in English. Please note that PLOS is an English-language publisher, so we require data sets to be provided in English as well. Please upload an English-language version of your data set.

This will also allow us to determine if your data follows PLOS standards per our Data Availability policy here: [https://journals.plos.org/plosone/s/data-availability](https://track.editorialmanager.com/CL0/https:%2F%2Fjournals.plos.org%2Fplosone%2Fs%2Fdata-availability/1/010f019a913a1680-8cd1e0e0-e977-4aa1-bc15-eb624c9dccca-000000/QcDluUYfqAMS60ioM1-1rvYCndzUsCMlfl6zpAS_DaI=237)

**We have uploaded an English-language version of our data set.**

8. We note that there is identifying data in the Supporting Information file “student award information(english ).xlsx”. Due to the inclusion of these potentially identifying data, we have removed this file from your file inventory. Prior to sharing human research participant data, authors should consult with an ethics committee to ensure data are shared in accordance with participant consent and all applicable local laws.

**We have removed all personal information in the Supporting Information file “student award information(english ).xlsx” to ensure data are shared in accordance with participant consent and all applicable local laws.**

Data sharing should never compromise participant privacy. It is therefore not appropriate to publicly share personally identifiable data on human research participants. The following are examples of data that should not be shared:

-Name, initials, physical address

-Ages more specific than whole numbers

-Internet protocol (IP) address

-Specific dates (birth dates, death dates, examination dates, etc.)

-Contact information such as phone number or email address

-Location data

-ID numbers that seem specific (long numbers, include initials, titled “Hospital ID”) rather than random (small numbers in numerical order)

Data that are not directly identifying may also be inappropriate to share, as in combination they can become identifying. For example, data collected from a small group of participants, vulnerable populations, or private groups should not be shared if they involve indirect identifiers (such as sex, ethnicity, location, etc.) that may risk the identification of study participants.

Additional guidance on preparing raw data for publication can be found in our Data Policy (<https://journals.plos.org/plosone/s/data-availability#loc-human-research-participant-data-and-other-sensitive-data>) and in the following article: <http://www.bmj.com/content/340/bmj.c181.long>.

Please remove or anonymize all personal information (<specific identifying information in file to be removed>), ensure that the data shared are in accordance with participant consent, and re-upload a fully anonymized data set. Please note that spreadsheet columns with personal information must be removed and not hidden as all hidden columns will appear in the published file.

**We have removed all personal characteristic-related information from the supporting information file named "Student Award Information".**

**Comments to the Author**
1. Is the manuscript technically sound, and do the data support the conclusions?
The manuscript must describe a technically sound piece of scientific research with data that supports the conclusions. Experiments must have been conducted rigorously, with appropriate controls, replication, and sample sizes. The conclusions must be drawn appropriately based on the data presented.

Reviewer #1: Partly

Reviewer #2: Yes

2. Has the statistical analysis been performed appropriately and rigorously?

Reviewer #1: No

Reviewer #2: Yes

**We have incorporated detailed statistical analysis content.**

3. Have the authors made all data underlying the findings in their manuscript fully available?
The [PLOS Data policy](http://track.editorialmanager.com/CL0/http:%2F%2Fwww.plosone.org%2Fstatic%2Fpolicies.action%23sharing/1/010f019a913a1680-8cd1e0e0-e977-4aa1-bc15-eb624c9dccca-000000/aiZO2fHYIPmfawIBCvp6IOeQlyEULM2wisjL6M31yE8=237) requires authors to make all data underlying the findings described in their manuscript fully available without restriction, with rare exception (please refer to the Data Availability Statement in the manuscript PDF file). The data should be provided as part of the manuscript or its supporting information, or deposited to a public repository. For example, in addition to summary statistics, the data points behind means, medians and variance measures should be available. If there are restrictions on publicly sharing data—e.g. participant privacy or use of data from a third party—those must be specified.

Reviewer #1: Yes

Reviewer #2: Yes

4. Is the manuscript presented in an intelligible fashion and written in standard English?
PLOS ONE does not copyedit accepted manuscripts, so the language in submitted articles must be clear, correct, and unambiguous. Any typographical or grammatical errors should be corrected at revision, so please note any specific errors here.

Reviewer #1: No

Reviewer #2: Yes

**We have thoroughly revised the expressions in the paper to ensure the use of standard English.**

5. Review Comments to the Author
Please use the space provided to explain your answers to the questions above. You may also include additional comments for the author, including concerns about dual publication, research ethics, or publication ethics. (Please upload your review as an attachment if it exceeds 20,000 characters)

Reviewer #1:

1. This instructional strategy is innovative and has the potential for broader application. While the paper is a valuable contribution, some revisions are necessary before it can be considered for publication.

**We have made conscientious revisions based on the reviewer's comments.**

2. Please provide a brief introduction to the BOPPPS model earlier in the article.

**We have added a brief introduction to the BOPPPS model in 2.1.**

3. Please provide evidence of the reliability or maybe validity for the survey scale used in the study.

**We have added reliability information in 4.2.**
4. Please provide more detailed statistical values, such as the paired Cohen’s d and 95% CIs.

**We have added paired Cohen’s d and 95% CIs information in table 3.**

5. The paper would be strengthened by including the explicit results from the qualitative analysis, which the authors state was conducted in Section 3.3.4.

**The original paper did include content on qualitative analysis, but it was not presented clearly enough. Therefore, we have reorganized the subsections in Chapter 4 of the paper, with Section 4.2 dedicated to qualitative analysis.**

6. A thorough language and technical edit is required prior to resubmission to address several errors in the text. For example: Reference 6 appears to be missing from the in-text citations, and the definition provided for the abbreviation VR is incorrect.

**The indexing error in Reference 6 has been corrected, and all spelling mistakes and technical errors in the paper have been rectified.**

**------------------------------------------------------------------------------------------**

Reviewer #2: Overview
This study describes an effective and relevant approach to teaching Digital Signal Processing by integrating sustainability themes into the learning process. The combination of the BOPPPS model and CIBL framework promotes student participation, critical thinking, and understanding of the connection between engineering education and sustainable development. The topic aligns with current trends in educational innovation and applied learning.

Major Strengths
• Clear and well-motivated instructional design linking DSP education with sustainability goals.
• Ethical approval and student consent are clearly documented.
• Balanced use of qualitative and quantitative assessment methods.
• Open sharing of anonymized data and transparent research procedures.
• Logical structure and clear presentation of results.

Points for Improvement (Minor Revisions)
1. Provide basic demographic information such as sample size, gender distribution, and response rate for clarity.

**We have added the sample size, gender distribution, and response rate in 4.2.1.**

2. Add statistical details such as confidence intervals or effect sizes to show the significance of improvements.

**We have added confidence intervals and effect sizes in table 3.**

3. Improve figure and table captions so that they are self-explanatory.

**We have improve the information in all figures and table titles.**

4. Discuss briefly how the proposed model can be applied to other engineering or online teaching contexts.

**Section 4.4 has been supplemented with a brief discussion on the application of the proposed solution in other engineering-related courses.**

5. Review the manuscript for minor grammatical corrections and consistent terminology.
**We have corrected the grammatical errors and inconsistencies in terminology usage.**

Ethics and Data Availability
Ethical approval (LLSC-2024-082001) and written consent were properly obtained. All data are available in the supplementary materials and meet the PLOS ONE open-data requirements. No ethical concerns were found.

Overall Recommendation
Minor Revision. The paper is methodologically sound, ethically compliant, and presents a valuable contribution to sustainable engineering education. Only minor additions to statistical reporting and formatting are needed before publication.
Reviewer Certification
This review was written independently and reflects my professional evaluation of the manuscript. It complies with PLOS ONE and COPE Ethical Guidelines for Peer Reviewers.

6. PLOS authors have the option to publish the peer review history of their article ([what does this mean?](https://track.editorialmanager.com/CL0/https:%2F%2Fjournals.plos.org%2Fplosone%2Fs%2Feditorial-and-peer-review-process%23loc-peer-review-history/1/010f019a913a1680-8cd1e0e0-e977-4aa1-bc15-eb624c9dccca-000000/b54GXOmMhGf9pLsw4yGCSQfMSsbR5A_gKPXlFACHeio=237)). If published, this will include your full peer review and any attached files.
If you choose “no”, your identity will remain anonymous but your review may still be made public.
**Do you want your identity to be public for this peer review?** For information about this choice, including consent withdrawal, please see our [Privacy Policy](https://track.editorialmanager.com/CL0/https:%2F%2Fwww.plos.org%2Fprivacy-policy/1/010f019a913a1680-8cd1e0e0-e977-4aa1-bc15-eb624c9dccca-000000/Nv1UQJtD0pd16ES47IAfqI1V1iPSgDZ9j4FKPA3MXTc=237).

Reviewer #1: **Yes:** Chih-Cheng Lin

Reviewer #2: **Yes:** Sara Goze

[NOTE: If reviewer comments were submitted as an attachment file, they will be attached to this email and accessible via the submission site. Please log into your account, locate the manuscript record, and check for the action link "View Attachments". If this link does not appear, there are no attachment files.]

To ensure your figures meet our technical requirements, please review our figure guidelines: <https://journals.plos.org/plosone/s/figures>

You may also use PLOS’s free figure tool, NAAS, to help you prepare publication quality figures: <https://journals.plos.org/plosone/s/figures#loc-tools-for-figure-preparation>.

NAAS will assess whether your figures meet our technical requirements by comparing each figure against our figure specifications.
